# PKN1 kinase-negative knock-in mice develop splenomegaly and leukopenia at advanced age without obvious autoimmune-like phenotypes

**DOI:** 10.1038/s41598-019-50419-2

**Published:** 2019-09-27

**Authors:** Salman Mahmud Siddique, Koji Kubouchi, Yuka Shinmichi, Nana Sawada, Reiko Sugiura, Yasushi Itoh, Shunsuke Uehara, Kanae Nishimura, Shunsuke Okamura, Hiroyuki Ohsaki, Shingo Kamoshida, Yusuke Yamashita, Shinobu Tamura, Takashi Sonoki, Hiroshi Matsuoka, Tomoo Itoh, Hideyuki Mukai

**Affiliations:** 1 Graduate School of Medicine, Kobe University, Kobe, 650–0017 Japan; 2 Laboratory of Molecular Pharmacogenomics, School of Pharmaceutical Sciences, Kindai University, 3-4-1, Kowakae, Higashi-Osaka, 577- 8502 Japan; 3 Department of Pathology, Shiga University of Medical Science, Otsu, Shiga Japan; 4 Department of Biochemistry, Matsumoto Dental University, Shiojiri, Nagano 399-0781 Japan; 5 Laboratory of Pathology, Department of Medical Biophysics, Kobe University Graduate School of Health Sciences, 7-10-2 Tomogaoka, Suma, Kobe, Hyogo 654-0142 Japan; 6 Department of Hematology/Oncology, Wakayama Medical University, Wakayama, Japan; 7 Division of Medical Oncology and Hematology, Kobe University Hospital, Kobe, Hyogo Japan; 8 Department of Diagnostic Pathology, Kobe University Hospital, Kobe, Hyogo 650-0017 Japan; 9 Biosignal Research Center, Kobe University, Kobe, 657- 8501 Japan

**Keywords:** Kinases, Autoimmunity, Spleen

## Abstract

Protein kinase N1 (PKN1) knockout (KO) mice spontaneously form germinal centers (GCs) and develop an autoimmune-like disease with age. Here, we investigated the function of PKN1 kinase activity *in vivo* using aged mice deficient in kinase activity resulting from the introduction of a point mutation (T778A) in the activation loop of the enzyme. PKN1[T778A] mice reached adulthood without external abnormalities; however, the average spleen size and weight of aged PKN1[T778A] mice increased significantly compared to aged wild type (WT) mice. Histologic examination and Southern blot analyses of spleens showed extramedullary hematopoiesis and/or lymphomagenesis in some cases, although without significantly different incidences between PKN1[T778A] and WT mice. Additionally, flow cytometry revealed increased numbers in B220^+^, CD3^+^, Gr1^+^ and CD193^+^ leukocytes in the spleen of aged PKN1[T778A] mice, whereas the number of lymphocytes, neutrophils, eosinophils, and monocytes was reduced in the peripheral blood, suggesting an advanced impairment of leukocyte trafficking with age. Moreover, aged PKN1[T778A] mice showed no obvious GC formation nor autoimmune-like phenotypes, such as glomerulonephritis or increased anti-dsDNA antibody titer, in peripheral blood. Our results showing phenotypic differences between aged *Pkn1*-KO and PKN1[T778A] mice may provide insight into the importance of PKN1-specific kinase-independent functions *in vivo*.

## Introduction

PKN1 (also known as PKNα or PRK1) is one of three PKN isoforms (PKN1, PKN2, and PKN3) derived from different genes in mammals^1^ and activated by fatty acids and phospholipids, as well as following cleavage by a caspase-3-like protease^2–6^. Additionally, PKN1 is an effector protein kinase of Rho family GTPases, such as RhoA, RhoB, RhoC, and Rac 1, in mammalian tissues^7–13^. PKN1 is expressed ubiquitously and involved in various functions, including cell-cell adhesion^14,15^, cell migration^16–19^, vesicle transport^20,21^, cell survival^22–26^, cell cycle regulation^18,27–31^, transcriptional regulation^32–36^, tumorigenesis^19,32,33,37,38^, myogenic differentiation^39^, parallel fiber-Purkinje-cell synapse formation in the cerebellum^40^, and pyrin inflammasome formation^41,42^. In some cases, PKN1 reportedly plays important roles independent of its kinase activity, such as activation of phospholipase D1^43^, scaffolding for p38γ mitogen-activated protein kinase signaling^44^, and increasing the survival of cardiac muscle cells following ischemia/reperfusion^24^. Therefore, we speculate that the absence of PKN1 might result in a complex phenotype attributed to the loss of both kinase-dependent and -independent functions. Yasui *et al*.^45^ generated *Pkn1*-knockout (KO) mice that display an appearance comparable to that of the control and do not exhibit defects in lymphocyte development within 12 weeks of age; however, germinal centers (GCs) form spontaneously in the spleen at >30 weeks of age, even in the absence of immunization or infection, showing an autoimmune-like disease characterized by autoantibody production and glomerulonephritis. These phenotypes associated with aged *Pkn1*-KO mice suggest an indispensable function for physiologically appropriate GC B cell survival and selection^45^. On the other hand, we previously investigated the kinase activity-dependent role of PKN1 *in vivo* by generating *Pkn1* homozygous T778A-knock-in (PKN1[T778A]) mice, lacking PKN1 kinase activity via disruption of the catalytic domain^46^. We found that mice aged 7 to 9 weeks exhibited selective decreases in the number of lymphocytes in peripheral blood and increases in this number in the spleen and lymph nodes in the absence of apparent abnormality in lymphocyte-cell development in the bone marrow and thymus^46^. This phenotype appeared to originate via defective lymphocyte egress from secondary lymphoid organs and was supported by significantly lower chemotaxis of lymphocytes deficient in kinase activity toward chemokines, such as sphingosine 1-phosphate (S1P) relative to that observed in wild-type (WT) cells *in vitro*^46^. These data suggest that PKN1 kinase activity is critical for lymphocyte trafficking in young mice; however, the long-term physiological role of this activity and related to the eventual phenotype in aged PKN1[T778A] mice have not been analyzed. In the present study, we performed phenotypic analysis of aged PKN1[T778A] mice and discussed potential differences in the phenotypes of aged PKN1[T778A] and previously reported *Pkn1*-KO mice.

## Results

### PKN1[T778A] mice show enlarged spleens at an advanced age

**Figure 1:**
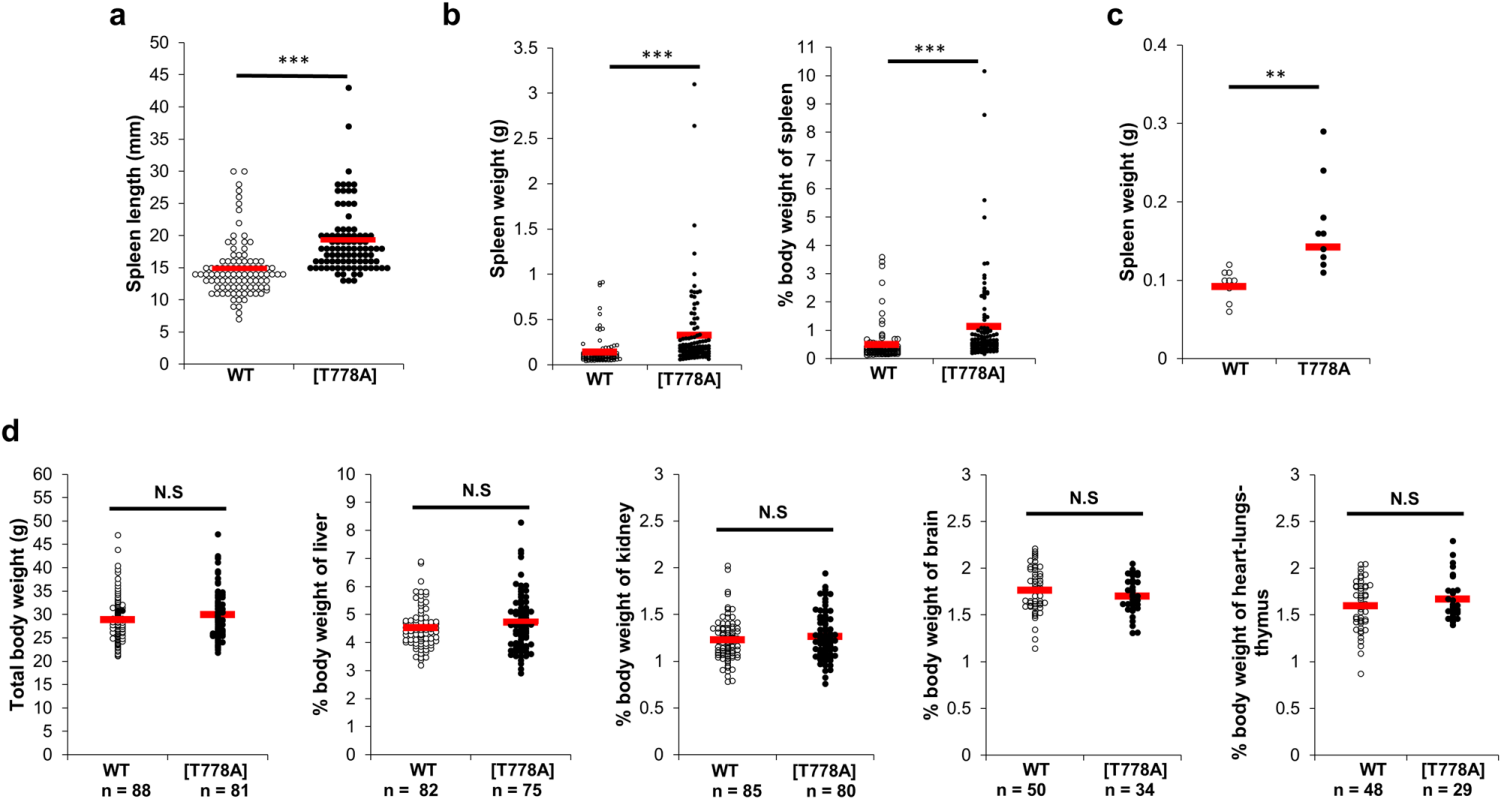
Enlargement of spleen in aged PKN1[T778A] mice. (**a**) Comparison of splenic length between aged WT and PKN1[T778A] mice. n = 98, WT; 92, PKN1[T778A]. Data were analyzed by Mann-Whitney U test. ***P < 0.001. (**b**) Comparison of actual spleen weight (g) and % body weight of spleen between aged WT and PKN1[T778A] mice. n = 100, WT; 103, PKN1[T778A]. Data were analyzed by Mann-Whitney U test. ***P < 0.001. (**c**) Comparison of spleen weight (g) between WT and PKN1[T778A] mice of 40–50 weeks age. n = 9. Data were analyzed by unpaired *t*-test. **P < 0.01. (**d**) Comparison of total body weight (g) and % body weight of other major organs between aged WT and PKN1[T778A] mice. Data were analyzed by unpaired *t*-tests. NS, not significant.

To determine the long-term effects of PKN1 kinase deficiency in mice, we examined the organs of PKN1[T778A] and control WT mice aged >1 year (male and female) [Table 1; WT male (*n* = 54), female (*n* = 46); PKN1[T778A] male (*n* = 53), female (*n* = 50)]. Measurements of major organs (size and weight) between mutant and control mice revealed a statistically significant enlargement of the spleen in PKN1[T778A] mice (Fig. 1a,b) relative to WT mice, with an average >2-fold increase in the spleen weight of PKN1[T778A] mice (0.33 g/1.13% of body weight) relative to WT mice (0.14 g/0.48% of body weight). In addition, PKN1[T778A] mice aged 40–50 weeks also showed a significantly larger spleen than WT mice (Fig. 1c). No significant difference in size and weight of other major organs such as the liver, kidney, heart, lungs, and brain were observed between aged WT and aged PKN1[T778A] mice (Fig. 1d). We observed a higher incidence of enlarged lymph nodes (inguinal and mesenteric) in aged PKN1[T778A] mice relative to that in aged WT mice, as well as an enlarged spleen (Table 1).

**Table 1:** Secondary lymphoid organs in aged mice. ILN, inguinal lymph node; MLN, mesenteric lymph node.

	Number of mice	Spleen >1% body weight	Large ILN	Large MLN
WT	100	8 (8%)	10 (10%)	11 (11%)
PKN1[T778A]	103	27 (26.2%)	19 (18.5%)	21 (20.4%)

### Extramedullary hematopoiesis is not a major cause of characteristic spleen enlargement in aged PKN1[T778A] mice

**Figure 2:**
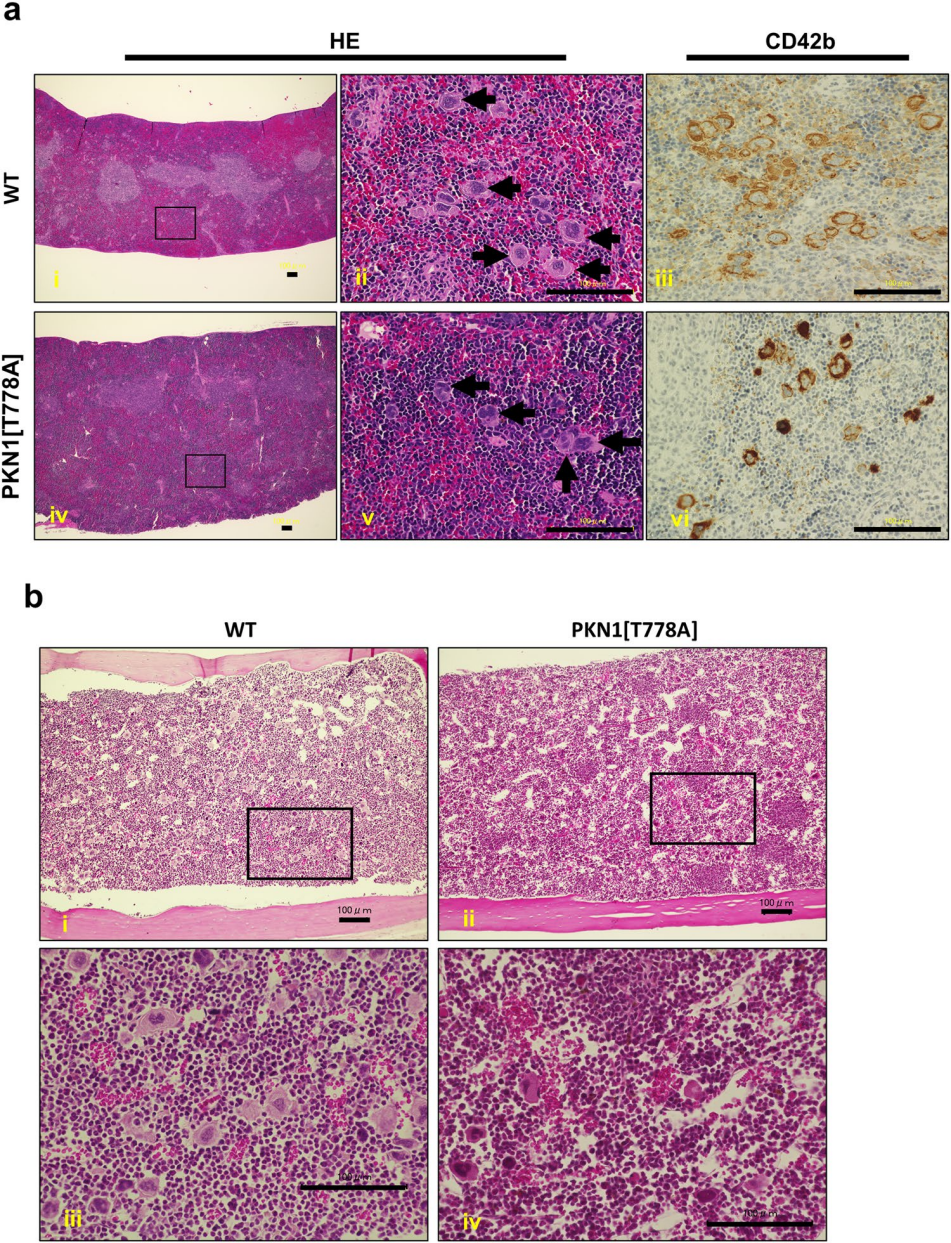
Extramedullary hematopoiesis in aged mice. (**a**) Histological analysis of spleen. Representative observation from samples of spleens of aged mice are shown (i, ii, iii: WT; iv, v, vi: PKN1[T778A]). HE, hematoxylin & eosin staining; CD42b, immunostaining for CD42b to detect megakaryocytes. ii and v are higher magnifications of the boxed area of i and iv respectively. Arrows indicate the presence of megakaryocytes. Scale bar: 100 μm. (**b**) Representative hematoxylin & eosin staining of femoral sections from an aged WT and PKN1[T778A] mouse pair (i, iii: WT; ii, iv: PKN1[T778A]). Scale bar: 100 μm.

To clarify the cause of splenic enlargement, we first performed hematoxylin and eosin (H&E) staining of sections of larger spleens from both aged PKN1[T778A] and aged WT mice and compared them with normal spleens from young WT mice. We observed expansion of the red pulp along with increased cellularity in some cases in both aged PKN1[T778A] and aged WT mice (Fig. 2a and Supplementary Tables S1 and S2; WT: B522, B577, WT1a, B200, B471, B116, and B780; PKN1[T778A]: U664, U691, T433, T129, B723, C165, B416, B529, B970, and B415). Additionally, megakaryocytes, confirmed via anti-CD42b antibody staining of spleen sections (Fig. 2a) clearly increased in these mice (Supplementary Tables S1 and S2). Expansion of splenic red pulp along with increased cellularity and an abundance of megakaryocytes represent key features of enhanced extramedullary hematopoiesis in the spleen^47,48^, with extramedullary hematopoiesis, in general, representing a valid cause of an enlarged spleen^49–51^. Therefore, we speculate that PKN1[T778A] mice may harbor susceptible phenotypes promoting extramedullary hematopoiesis, despite extramedullary hematopoiesis-like histological features were recorded in both aged PKN1[T778A] and aged WT mice. Peripheral blood analysis did not show “anemia” in aged PKN1[T778A] mice, which is frequently linked to the enhanced extramedullary hematopoiesis (Supplementary Table S3). Because aging-associated osteomyelosclerosis-related bone-marrow hypoplasia leads to enhanced extramedullary hematopoiesis^48^, and PKN3, which is a PKN1 isoform, is essential for bone resorption by osteoclasts^52^, we then investigated whether aged PKN1[T778A] mice display ossification and bone-marrow hypoplasia. H&E staining of femoral sections revealed neither bone-marrow hypoplasia nor trabeculae formation within the bone-marrow space of aged PKN1[T778A] as well as control WT mice (Fig. 2b). Moreover, we could not detect any notable difference in bone volume between PKN1[T778A] and WT mice according to micro-computed tomography (CT) analysis of femora and fifth lumbar vertebrae from aged mice, further supporting the histological findings (Supplementary Fig. S1). Taken together, these results suggest that enhanced extramedullary hematopoiesis is not a specific feature of aged PKN1[T778A] mice.

### Incidence of lymphomagenesis in aged PKN1[T778A] mice does not differ significantly from that in aged WT mice

**Figure 3:**
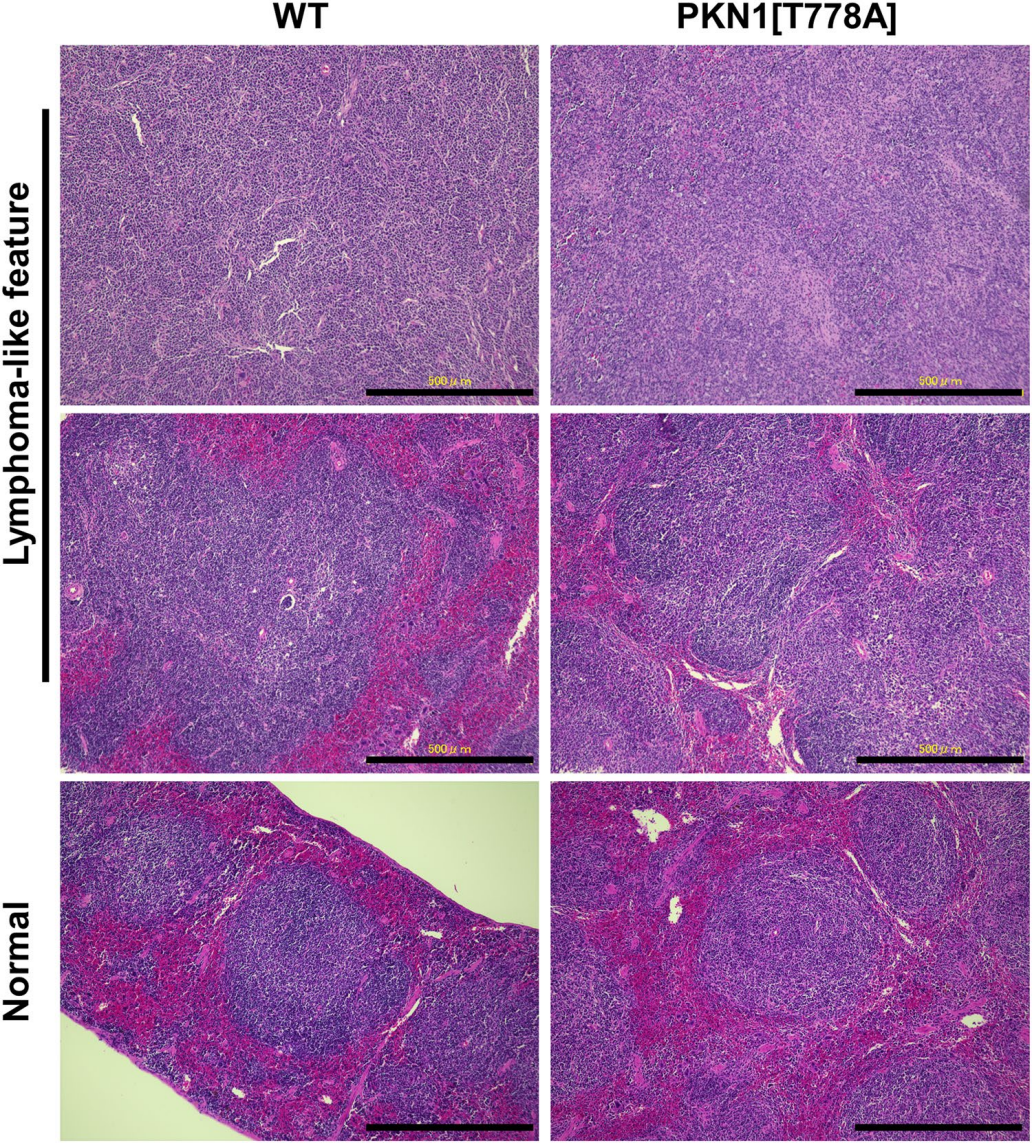
Comparable incidence of lymphomagenesis between aged PKN1[T778A] and WT mice. Representative observation of HE staining from samples of spleen with distorted follicular structure (upper and middle panel) and normal follicular structure (lower panel) from aged WT and PKN1[T778A] mice. Scale bar: 500 μm.

In most lymphoid-cell malignancies, such as lymphomas, clonal proliferation of B and/or T lymphocytes gives rise to the expansion of secondary lymphoid organs, such as the spleen or lymph nodes^53^. H&E staining of the spleen sections of mice aged >60 weeks, revealed a considerable number of PKN1[T778A] and WT mice with large spleens also displaying distorted follicular structure determined by either the absence of or a wide marginal zone of the follicles (Fig. 3; Supplementary Tables S1 and S2; 9 of 15 WT and 18 of 23 PKN1[T778A] mice). Some of these structural distortions were associated with either expansion of the white-pulp area or an increased number of follicles (Supplementary Tables S1 and S2; WT: C102, B516, C109, WT3a, C171, C069, and C690; PKN1[T778A]: U793, U791, U690, T129, S106, B969, B657, B742, B530, and B418). Because PKN1 reportedly inhibits transforming activity of Akt and enhances the activation-induced cell death of B lymphocytes^45^, long-term deficiency in PKN1 activity appears sufficient to induce lymphomagenesis. Therefore, we examined whether incidence of lymphomagenesis is clearly higher in aged PKN1[T778A] mice than in aged WT mice. Enhanced monoclonal rearrangement of the *immunoglobulin heavy chain (IgH)* gene J region and *T cell receptor (Tcr)* gene JB2 region commonly occurs in most B and T cell lymphomas, respectively, and indicates the clonality of malignant lymphoid proliferation as a hallmark for confirming lymphoma^54–56^. Therefore, we performed Southern blot analysis using mouse splenic DNA digested with different restriction enzymes and hybridized with radioisotope-labeled pMJH4 and TCRB probes in order to detect the rearrangement of the *Igh* J region and/or *Tcr* JB2 region, respectively (Supplementary Fig. S2). Twelve of the 25 aged PKN1[T778A] mice with enlarged spleens showed enhanced monoclonal rearrangement of the *Igh* J region and one of the *Tcr* JB2 region (Table 2 and Supplementary Fig. S2). For the WT mice, we found that 6 of 15 mice showed enhanced rearrangement of the *Igh* J region and 2 showed rearrangement of the *Tcr* JB2 region (Table 2 and Supplementary Fig. S2). These results suggest that the incidence of lymphomagenesis in aged PKN1[T778A] mice is similar to that in aged WT mice. Notably, the incidence of lymphomagenesis appeared independent of the relative size of the spleens tested (Table 2).

**Table 2:** Results of Southern blot analysis of splenic DNA from aged mice.

Mouse ID.		Basic information			Southern blotting			
					pMJH4		TCRB	
		Age (W)	Gender	Sp.wt. (g)	Result	Remark	Result	Remark
WT	C102	64	F	0.91	+	for *Eco*RI, *Bam*HI, *Bgl*II	−	
	B522	96	F	0.90	−		−	
	B516	94	M	0.90	+	for *Bam*HI	−	
	B577	92	F	0.63	+	for *Bam*HI	−	
	C109	64	F	0.43	−		−	
	WT 1a	98	F	0.40	−		−	
	B200	110	M	0.39	−		−	
	B471	94	M	0.27	−		−	
	WT 3a	98	F	0.22	+	for *Hin*dIII	+	for *Hin*dIII
	B485	85	M	0.21	+	for *Eco*RI, *Hin*dIII, *Bgl*II	−	
	B116	118	M	0.19	−		−	
	C171	61	F	0.18	+	for *Hin*dIII, *Bam*HI	−	
	B609	82	M	0.15	−		−	
	C069	67	F	0.14	−		+	for *Eco*RI
	C690	70	F	0.13	−		−	
PKN1[T778A]	U791	88.9	F	1.23	+	for *Eco*RI, *Bam*HI, *Bgl*ll	−	
	U664	88.3	F	1.00	+	for *Eco*RI	−	
	B497	90	M	0.87	−		−	
	U691	98.4	F	0.81	+	for *Eco*RI	−	
	R678	90	F	0.80	+	for *Eco*RI, *Hin*dIII	−	
	T129	81	F	0.75	+	for *Eco*RI	−	
	S106	90	M	0.68	+	for *Eco*RI	+	for *Hin*dIII
	B969	74	F	0.62	+	for *Eco*RI, *Bam*HI	−	
	B723	82	M	0.51	−		−	
	B657	76	F	0.33	+	for *Eco*RI	−	
	C165	61	F	0.32	−		−	
	B416	98	M	0.29	−		−	
	B529	84	F	0.29	−		−	
	B970	74	F	0.27	−		−	
	R675	90	F	0.26	−		−	
	B415	98	M	0.23	−		−	
	C081	65	F	0.22	+	for *Eco*RI	−	
	C085	65	F	0.21	−		−	
	B331	101	M	0.21	−		−	
	A771	77	M	0.20	+	for *Eco*RI	−	
	B742	76	M	0.20	+	for *Eco*RI	−	
	B530	84	F	0.19	−		−	
	B418	89	F	0.19	−		−	
	C167	61	F	0.19	−		−	
	C088	65	F	0.18	+	for *Eco*RI	−	

Total 15 WT and 25 PKN1[T778A] mice were analyzed.+, enhanced clonal rearrangement; −, no enhanced clonal rearrangement; W, week; Sp.wt.(g), spleen weight in gram.

### Matured leukocyte populations increase in the spleen and decrease in the peripheral blood of aged PKN1[T778A] mice

**Figure 4:**
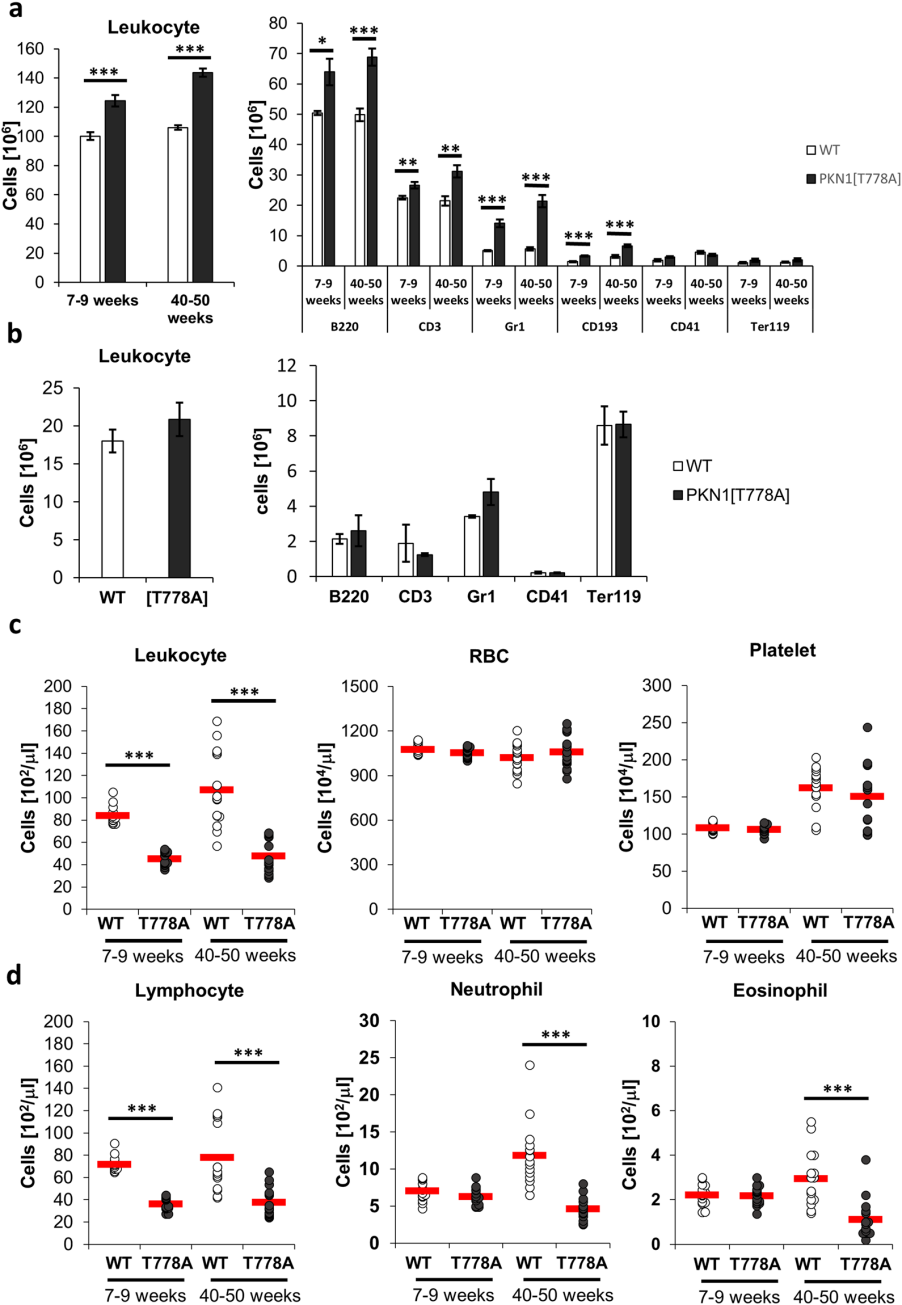
Cell count of spleen, bone marrow and peripheral blood from WT and PKN1[T778A] mice. (**a**) Spleen (n = 5) and (**b**) bone marrow (n = 3) cells of WT and PKN1[T778A] mice were characterized by flow cytometric analysis using fluorophore conjugated antibodies against indicated cell markers. Bone marrow cells were counted as the number of cells per femur. (**c**) Peripheral blood count (n = 15) and (**d**) leukocyte cellularity in the peripheral blood (n = 15). Data were analyzed by unpaired *t*-tests. *P < 0.05, **P < 0.01, ***P < 0.001.

Southern blot analysis of splenic cells showed enhanced monoclonal rearrangements in some mice aged >50 weeks, as described above. Therefore, we focused on phenotypic analysis of spleens from mice aged 40 to 50 weeks in order to avoid the effect of lymphomagenesis. As shown in Fig. 1c, PKN1[T778A] mice in this age range showed significant enlargement of the spleen relative to their size in WT mice. To investigate the possible expansion of specific splenic cell populations accountable for this enlargement, we performed flow cytometric analysis using splenocytes from mice aged 40 to 50 weeks. We found that total leukocyte count was significantly elevated in the PKN1[T778A] mouse spleen, with B220^+^ (B cell marker), CD3^+^ (T cell marker), Gr1^+^ and CD193^+^ (granulocyte markers) cells especially increased relative to levels in WT mice (Fig. 4a). Flow cytometric analysis of bone-marrow cells showed no significant difference in cell count between aged PKN1[T778A] and aged WT mice, although we observed a tendency of increased number of Gr1^+^ cells in PKN1[T778A] mice relative to WT mice (Fig. 4b). On the other hand, analysis of circulating peripheral blood collected from the facial vein revealed a significantly lower number of leukocytes in both young and aged PKN1[T778A] mice than in WT mice, despite comparable numbers of erythrocytes and platelets (Fig. 4c). Among the peripheral leukocytes, absolute counts of lymphocytes were significantly lower in young and aged PKN1[T778A] mice relative to WT mice (Fig. 4d). Counts of neutrophils and eosinophils were normal in young mice, although aged PKN1[T778A] mice showed markedly reduced counts of these cell populations relative to WT mice (Fig. 4d). To determine whether abnormality in immune cell distribution affects the antigen-specific immune response in PKN1[T778A] mice, we subcutaneously injected ovalbumin (OVA) in mice twice with 1-week interval and compared the elevation of OVA-specific IgG and IgM production between WT and PKN1[T778A] mice. One week after the second injection, we measured serum IgG and IgM titers in WT and PKN1[T778A] mice. As shown in Supplementary Fig. S3, no significant difference in the titer of OVA-specific serum IgG and IgM was observed between WT and PKN1[T778A] mice.

### Autoimmune disease-like phenotypes are not observed in aged PKN1[T778A] mice

**Figure 5:**
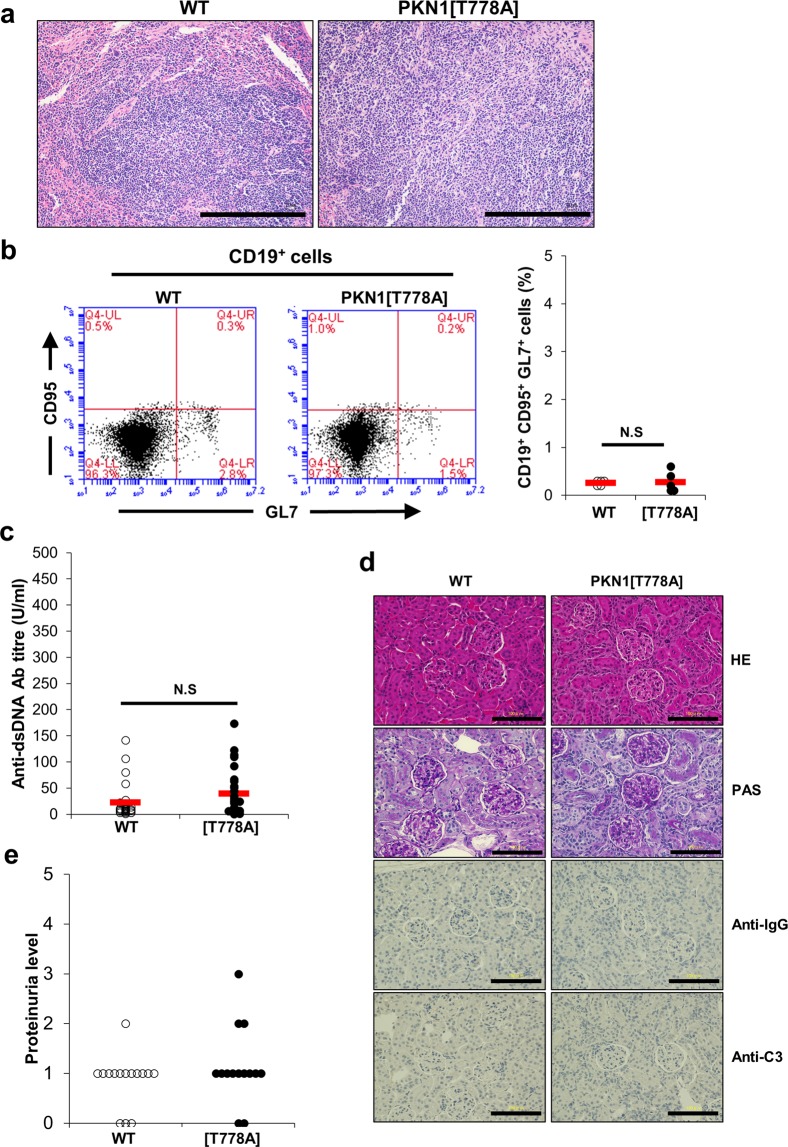
Absence of autoimmune-like phenotypes in aged PKN1[T778A] mice. (**a**) Representative HE staining of spleen sections from WT and PKN1[T778A] mice >60 weeks of age both showing absence of germinal center (GC) within the follicles. Scale bar: 500 μm. (**b**) Flow cytometric analysis of GC B cells from splenocytes that were stained with CD19, GL7 and CD95. Statistical analysis of CD95^+^ GL7^+^ B cells in splenic CD19^+^ B cells. Data were analyzed by unpaired *t*-test. n = 5. NS, not significant. (**c**) Serum Anti-dsDNA titer in >1 year aged mice evaluated by ELISA. Data were analyzed by Mann-Whitney U test. n = 27, WT; 28, PKN1[T778A]. NS, not significant. (**d**) Absence of glomerulonephritis in aged PKN1[T778A] mice. Hematoxylin-eosin staining (HE, *Top*), Periodic acid-Schiff staining (PAS, *Second*), immune staining with anti-IgG antibodies (Anti-IgG, *Third*) and anti-C3 antibodies (Anti-C3, *Bottom*) of kidney sections from >1 year aged WT and PKN1[T778A] mice. Scale bar: 100 μm. (**e**) Urine protein level measured. Data represents experiment of Day-1 among 3 consecutive days. Data were analyzed by Mann-Whitney U test. n = 15, WT; 14, PKN1[T778A].

Marked leukopenia and spleen enlargement at an advanced age increase the likelihood of developing autoimmune disease^57,58^. A previous study reported that *Pkn1*-KO mice spontaneously form GCs at >30 weeks of age, develop an autoimmune-like disease characterized by proliferative glomerulonephritis at 6 months of age, and exhibit a substantial increase in serum level of anti-dsDNA antibodies at 1 year of age^45^. Therefore, we examined whether aged PKN1[T778A] mice suffer from these autoimmune-like phenotypes. GC formation was not evident in H&E-stained spleen sections from both PKN1[T778A] and WT mice at 40 weeks of age (Fig. 5a), and flow cytometric analysis using splenic cells showed that the CD19^+^ CD95^+^ GL7^+^ GC B cell fraction was small and comparable between PKN1[T778A] and WT mice at >30 weeks of age (Fig. 5b), suggesting no spontaneous or accelerated GC formation in aged PKN1[T778A] mice. Moreover, serum levels of anti-dsDNA antibodies in PKN1[T778A] mice >1 year of age were comparable with those in WT mice, with levels in both mouse groups much lower than the previously reported titer from *Pkn1*-KO mice (Fig. 5c)^45^. H&E and periodic acid‐Schiff (PAS) staining of kidney sections from mice >1 year of age revealed no increases in mesangial cell number or thickness of the basement membrane in glomeruli of PKN1[T778A] mice as compared with WT mice (Fig. 5d). Additionally, deposition of IgG and complement component 3 (C3) in mesangial regions were not evident in either aged PKN1[T778A] mice or aged WT mice according to immunohistochemistry (Fig. 5d). Because continuous proteinuria is characteristic feature of glomerulonephritis^59^, we collected urine samples for three consecutive days from PKN1[T778A] and WT mice aged >1 year and examined the protein. No significant difference was observed in protein content between aged PKN1[T778A] and aged WT mice (Fig. 5e and Supplementary Table 4). Because spontaneous recovery of kidney lesions as age advances appears unlikely, especially in autoimmune diseases, these results suggest that aged PKN1[T778A] mice do not significantly develop proliferative glomerulonephritis.

### Basal levels of Akt (S473) phosphorylation are not enhanced in splenocytes from PKN1[T778A] mice

**Figure 6:**
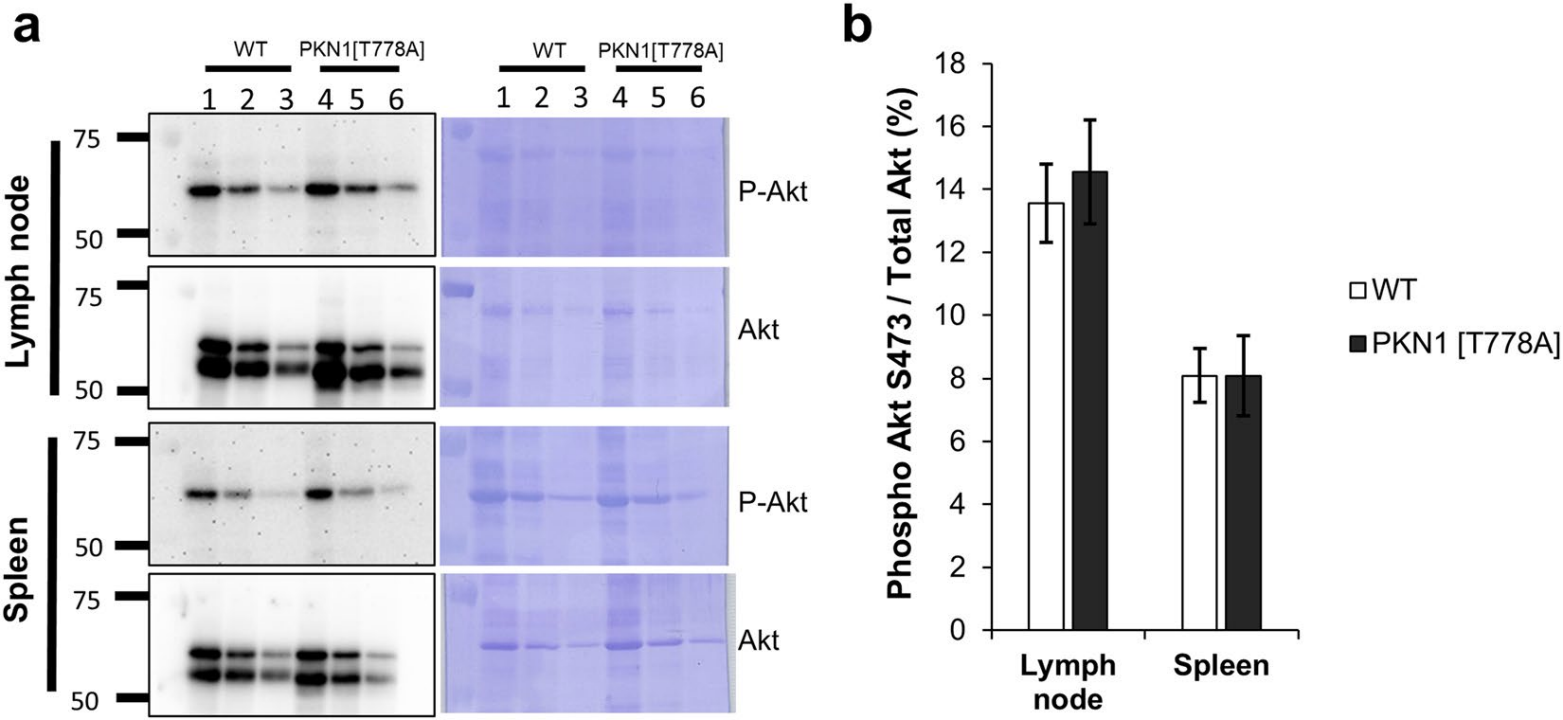
Measurement of phosphorylation of Akt at S473. (**a**) Representative image of immunoblotting (cropped images). Full length images are shown in Supplementary Fig. S5. Lane 1–3: WT samples; 1X, 2X and 4X dilutions. Lane 4–6: PKN1[T778A] samples; 1X, 2X and 4X dilutions. Molecular weight of Akt and P-Akt are 59 KDa and 60 KDa respectively. (**b**) Quantification of Akt phosphorylation at S473 position (relative to total Akt protein). Data represents average of six individual experiments. Data were analyzed by unpaired *t*-test.

A previous study suggested that PKN1 regulates B cell survival and selection, particularly in GCs, through down-regulation of Akt activity, and that B cells from *Pkn1*-KO mice harbor consistently enhanced “basal” levels of Akt (S473) phosphorylation in the absence of B cell-receptor stimulation^45^. To determine whether deficiency in PKN1 kinase activity upregulates Akt activity *in vivo*, we measured Akt (S473) phosphorylation levels by immunoblot analysis of splenic and lymph-node tissues from PKN1[T778A] and WT mice. To preserve the physiological status of the phosphorylated Akt protein, tissues were fixed using cold trichloroacetic acid (TCA)/acetone in order to avoid Akt dephosphorylation during sample preparation immediately after collection from mice under anesthesia. No significant differences in relative Akt (S473) phosphorylation levels were observed in PKN1[T778A] mice as compared with WT mice, in either splenic or lymph-node tissues (Fig. 6a,b). We also assayed Akt phosphorylation levels at T308 and T450, which are required for activation of Akt. As shown in Supplementary Fig. S4, relative Akt phosphorylation at T308 and T450 were comparable between WT and PKN1[T778A] mouse lymphoid tissues.

## Discussion

We observed spleen enlargement as a prominent phenotype in aged PKN1[T778A] mice. Extramedullary hematopoiesis and lymphomagenesis in spleens were observed in some cases; however, neither phenotype is likely to be an exclusive feature of aged PKN1[T778A] mice responsible for spleen enlargement. We previously reported that lymphocytes from PKN1[T778A] mice at aged 7–9 weeks show lower levels of chemotaxis toward chemokines, such as S1P, and have a tendency to accumulate in secondary lymphoid organs, likely owing to defective egress from these organs^46^. Consistent with this finding, lymphocyte count in the peripheral blood is significantly lower in PKN1[T778A] mice aged 7–9 weeks relative to that in control WT mice^46^. In the present study, B220^+^, CD3^+^, Gr1^+^, and CD193^+^ cell populations were significantly elevated in the spleen of aged PKN1[T778A] mice (Fig. 4a) in contrast to the marked decrease in leukocytes in peripheral blood from aged PKN1[T778A] mice (Fig. 4c,d). If PKN1 plays critical roles in the trafficking of not only lymphocytes but overall leukocytes, continued leukocyte accumulation might lead to the eventual enlargement of the spleen in aged PKN1[T778A] mice, although the general mechanism of splenic granulocyte turnover/trafficking has yet not been clarified^60^. It should be noted that the number of neutrophils and eosinophils in peripheral blood markedly decreased along with that of lymphocytes in PKN1[T778A] mice aged 40 weeks (Fig. 4c,d), although the numbers of neutrophils and eosinophils were within a normal range in PKN1[T778A] mice aged 7 to 9 weeks^46^. A potential reason for the reduced neutrophil count in the circulation in aged PKN1[T778A] mice is age-related reduction in neutrophil release from the bone marrow. After production and maturation, egress from the bone marrow requires neutrophil migration across the bone-marrow sinusoidal endothelium in an abluminal-to-luminal direction^61^. The bone-marrow sinusoidal endothelium is unique in that it constitutively expresses integrin ligands, such as vascular-cell adhesion molecule-1 and intercellular adhesion molecule-1^61^. Neutrophil migration across the bone marrow endothelium is facilitated by interaction of integrins, such as CD49D on neutrophils, with integrin ligands on bone-marrow endothelium^62,63^. Yuan *et al*. recently reported that *Pkn1*-KO impairs neutrophil-integrin activation and adhesion to endothelial cells, and that the mechanism involves polarization defects in RAB21 via reduced PKN1-mediated phosphorylation of rabphilin 3 A homolog, a RAB21 effector, in *Pkn1*-KO neutrophils^20^. Therefore, deficiency in PKN1 kinase activity might cause impaired adhesion of neutrophils to bone marrow endothelium, leading to reduced egress of neutrophils from the bone marrow and into circulation. This might be supported by the bone-marrow cell count in our aged PKN1[T778A] mice showing a tendency of increased number of Gr1^+^ cells as compared with that in control aged WT mice (Fig. 4b). A recent study reported that neutrophil proportions increased in the bone marrow and secondary lymphoid organs, such as the spleen, of elderly healthy C57BL/6J mice (aged 22–24 months) as compared with their younger counterparts^64^. This was suggested to be a result of chronic low-grade inflammation during aging (i.e., “inflammaging”) characterized by elevated levels of circulating proinflammatory cytokines^64^. PKN1 reportedly phosphorylates pyrin to inhibit formation of the pyrin inflammasome, which promotes inflammation by secreting pro inflammatory cytokines, such as interleukin (IL)-1β and IL-18^41,65^. Consistent with this finding, *Pkn1* knockdown using small-interfering RNA in lipopolysaccharide-primed bone-marrow-derived macrophages induces spontaneous IL-1β release^41^. In this context, it seems plausible that deficient PKN1 kinase activity renders mice vulnerable to inflammation, which might contribute to “inflammaging”-related neutrophil accumulation in the spleen of aged PKN1[T778A] mice. However, our aged PKN1[T778A] mice showed peripheral neutropenia, which is unlikely in cases of pyrin-inflammasome formation often associated with neutrophilia. It is possible that reduced pyrin phosphorylation owing to a lack of PKN1 kinase activity might be compensated by the presence of PKN2 in PKN1[T778A] mice.

*Pkn1*-KO mice show spontaneous GC formation and autoimmune-like disease characterized by autoantibody production and proliferative glomerulonephritis suggested as a consequence of hyperactivation of Akt in B cells^45^. In the present study, aged PKN1[T778A] mice showed neither obvious GC formation, increased anti-dsDNA-antibody titer in peripheral blood, nor signs of proliferative glomerulonephritis. What is the potential cause of this phenotypic difference, considering that both mice have null PKN1 kinase activity? Yasui *et al*. established *Pkn1*-KO mice using E14 ES cells derived from the 129/Ola mouse strain^45^, and our PKN1[T778A] mice were established using RENKA ES cells^66^ derived from C57BL/6N (Charles River). Yasui *et al*. reported that *Pkn1*-KO mice were backcrossed to C57BL/6 mice for 15 generations^45^, although the sub-strain name of C57BL/6 was not described. Therefore, phenotypic differences between *Pkn1*-KO and PKN1[T778A] mice might result from genetic variations between mouse strains/sub-strains, as genetic variations linking to differences in immune function and allergy have been reported even among C57BL/6 sub-strains^67^.

In our study, basal Akt (S473) phosphorylation levels in both splenic and lymph-node tissues from PKN1[T778A] mice were not elevated as compared with those from WT mice. Akt activity could remain within the normal range *in vivo* in PKN1[T778A] mice in contrast to that observed in *Pkn1*-KO mice, also possibly explaining the absence of these immunological phenotypes in PKN1[T778A] mice. *Pkn1*-KO mouse tissues lack PKN1 protein, whereas, PKN1 protein in PKN1[T778A] mouse tissues are present at ~50% their level in WT mice, although completely lacking kinase activity^46^. Even a lower concentration of PKN1 might efficiently control total Akt activity *in vivo* in a mechanism independent of kinase activity. A previous study reported that Akt activation requires phosphorylation at T308 in the activation loop by phosphoinositide-dependent kinase-1 (PDK1)^68^, and that phosphorylation of at S473 in the carboxyl-terminus mainly by mammalian target of rapamycin complex-2 (mTORC2)^69^, is required for maximal Akt activation^70^. Additionally, PKN1 is reportedly activated by binding to PDK1 via the PKN1 carboxyl-terminal region^71–73^. Moreover a PKN1 kinase-deficient mutant (K644E) remains capable of binding PDK1^71^; therefore it is possible that kinase-deficient PKN1[T778A] inhibits Akt kinase activity by interfering with the interaction of Akt with PDK1 *in vivo*, whereas this inhibition is abolished in *Pkn1*-KO mice^1^. A recent study demonstrated an interaction between PKN1 and mTORC2 at the turn motif of PKN1 and suggested PKN1 as an important effector of mTORC2^31^. Because Akt is also an mTORC2-effector protein, absence of PKN1 might enhance the Akt-mTORC2 interaction, leading to upregulated Akt (S473) phosphorylation in *Pkn1*-KO mice but not in PKN1[T778A] mice. Furthermore, Akt phosphorylations at T308 and S473 *in vivo* are not mutually exclusive^70^; therefore, affecting even one of these phosphorylation events likely results in different Akt activities between PKN1[T778A] and *Pkn1*-KO mice.

The phenotypic differences between aged *Pkn1*-KO and aged PKN1[T778A] mice might also be related to Rho GTPase interaction with PKN1. Interaction between PKN1 and PDK1 reportedly occurs in a Rho GTPase-dependent manner crucial for subsequent signaling pathways^72^. Rho GTPases (RhoA, RhoB, RhoC, and Rac1) mainly bind to the antiparallel coiled-coil domain 1 in the PKN1 amino-terminal regulatory region^7–12^. Because this region remains intact in PKN1[T778A], Rho GTPase binding to PKN1 is possible in PKN1[T778A] mice. A previous study reported that the endogenous and the GTPase activating protein (GAP)-stimulated GTPase activity of RhoA is inhibited by interaction with PKN1 and results in sustainment of the RhoA-PKN1 interaction^11^. Given the lack of PKN1 protein in *Pkn1*-KO mice, it is possible that unbound Rho GTPases normally targeted by PKN1 might interact with other Rho GTPase effector proteins, which contrasts with the process in PKN1[T778A] mice. Therefore, differences in Rho GTPase-mediated signaling between *Pkn1*-KO and PKN1[T778A] mice might explain the observed phenotypic differences. Additionally, active forms of Rho GTPases such as, RhoA and Rac1, can directly or indirectly interact with and activate the phosphoinositide 3-kinase-Akt signaling pathway^74–77^. Taken together, Rho GTPase and Akt-mediated signaling might play key roles in the different phenotypes observed between PKN1[T778A] and *Pkn1*-KO mice.

## Materials and Methods

### Animals

PKN1 kinase-negative knock-in mice (PKN1[T778A] mice) were generated as previously described^46^, and were backcrossed at least 10 times into the Charles River C57BL/6N background before phenotypic analysis. For all experiments, we used specific-pathogen-free mice that were ultimately compared with littermates or controls. This study was approved by the Institutional Animal Care and Use Committee of Kobe University (Permission numbers: 22-05-19, 25-07-01, HEN1-25-07-01, 30-06-02) and Kindai University (Permission number: KAPS-30-001), and was carried out according to the Kobe University Animal Experimentation Regulations and the Kindai University Animal Experimentation Regulations.

### Mouse dissection

Dissection was performed according to the standard protocol^78^. Briefly, mice were dissected after euthanasia using isoflurane inhalation. A portion of spleens and lymph nodes were frozen in liquid nitrogen immediately after dissection and preserved at −80 °C. The remaining organ samples were fixed with 4% paraformaldehyde (PFA) in 0.2 M phosphate buffer (PB) at 4 °C overnight, followed by replacing the PFA solution with 20% sucrose in 0.1% PB and maintaining at 4 °C until use.

### Antibodies

Monoclonal antibodies used for flow cytometric analysis of cell-surface antigens were as follow. Allophycocyanin (APC)-conjugated mouse/human anti-CD45R/B220 (clone RA3-6B2), APC-conjugated mouse anti-CD3 (clone 17A2), APC-conjugated mouse anti-Ly-6G/Ly-6C (Gr-1; clone RB6-8C5), APC-conjugated mouse anti-TER-119/erythroid cell, APC-conjugated mouse anti-CD41 (clone MWReg30), fluorescein isothiocyanate-conjugated mouse anti-CD193 (CCR3; clone J073E5), APC-conjugated mouse anti-CD34 (clone MEC14.7), and APC-conjugated mouse anti-Ly-6A/E (Sca-1; clone E13-161.7) were purchased from BioLegend (San Diego, CA, USA). The anti-CD42b antibody (SP219; ab183345) was purchased from Abcam (Cambridge, UK). Purified mouse anti-Akt (610860) was purchased from BD Biosciences (San Jose, CA, USA). Phosphorylated Akt (S473) (#9271) was purchased from Cell Signaling Technology (Danvers, MA, USA). For kidney immunostaining, goat anti-mouse C3 (V-20) (sc-14612) was purchased from Santa Cruz Biotechnology, and anti-goat horseradish peroxidase (HRP) polymer and anti-mouse HRP polymer were purchased from Nichirei Bioscience (Tokyo, Japan).

### Histological analysis

Histological staining of PFA-fixed spleen sections was performed as previously described^46^. Murine kidneys were fixed in 4% PFA and embedded in paraffin for histological analysis. Sections (3 µm) were cut and mounted on aminopropyltriethoxysilane-coated slides, and H&E staining was performed to assess histological features. PAS staining was performed to evaluate mesangial expansion. For immunohistochemical staining, endogenous peroxidase was inactivated by treatment with 0.3% hydrogen peroxide in methanol for 30 min. Heat-induced antigen retrieval was performed by incubating sections with 10 mM Tris base containing 1 mM ethylenediaminetetraacetic acid (pH 9.0) in a pressure cooker for 8 min. Nonspecific binding sites were blocked with 0.25% casein solution (Dako, Glostrup, Denmark) for 5 min. Sections for C3 analysis were incubated for 1 h with a goat polyclonal anti-C3 antibody (1:200), followed by incubation with an anti-goat HRP polymer for 1 h. Sections for IgG staining were incubated with an anti-mouse HRP polymer for 30 min. The reaction products were developed with a diaminobenzidine solution (Dako). Imaging was performed by using BZ-9000 All-in-one Fluorescence Microscope (Keyence).

### Southern blot analysis

Southern blot analysis was performed as previously described^79,80^. Briefly, after genomic DNA isolation by phenol/chloroform extraction and ethanol precipitation, 10 µg of DNA was digested with the indicated restriction enzymes (*Eco*RI, *Hin*dIII, *Bam*HI, and *Bgl*II), and electrophoresed on an 0.8% agarose gel, followed by transfer to a nylon membrane (Hybond -N; Amersham Bioscience, Little Chalfont, UK). Southern blots were hybridized with ^32^P-labeled either a 1.2-kb pMJH4 or a 2.3-kb TCR Jβ2 probe. Imaging was performed on a Typhoon FLA 9500 imager (GE Healthcare Life Sciences).

### Flow cytometry

Cell suspensions were prepared from bone marrow and spleens and treated with 1× ACK buffer (150 mM NH_4_Cl, 10 mM KHCO_3_, and 1 mM EDTA) to remove erythrocytes. After washing and resuspension with staining buffer (0.5% bovine serum albumin in phosphate-buffered saline), cells were labeled with the appropriate primary antibodies for 30 min on ice. After washing with staining buffer, cells were subjected to flow cytometry using an Accuri system (BD Biosciences), and data were analyzed using BD Accuri C6 software (BD Biosciences).

### Analysis of peripheral blood

Peripheral blood from mice was collected in anti-coagulant-coated tubes (BD Biosciences) from the facial vein by pricking with a 5.5-mm animal lancet (Goldenrod; Braintree Scientific, Braintree, MA, USA). Blood count was measured using an XN-1000V hematology analyzer (Sysmex Corp., Kobe, Japan).

### Immunization

Mice were subcutaneously immunized with 100 µg of OVA (Sigma-Aldrich, St. Louis, MO, USA) in the presence of Imject alum (Thermo Scientific, Rockford, IL, USA) in a 1:1 ratio (100 µL of Imject alum to 100 µL of immunogen) and a total volume of 200 µL per animal. Mice were immunized twice with a 1-week interval. One week after the second immunization, blood samples were collected from each individual mouse and serum was kept at −80 °C until used.

### Enzyme-linked immunosorbent assay (ELISA)

OVA-specific IgG and IgM were assayed using standard ELISA procedures. In short, polystyrene plates (Maxisorp, Denmark) were coated with OVA (10 mg/mL) overnight at 37 °C, washed twice with PBS, blocked with 1% BSA in PBS for 2 h at 37 °C, and incubated with serial dilutions of each mouse antiserum starting at 1/5. After incubation for 2 h at room temperature, the plates were washed 3 times with PBS with 0.05% Tween 20 (PBS-T) and incubated for 2 h at room temperature with horseradish peroxidase conjugated goat anti-mouse IgG and goat anti-mouse IgM at 1:4000 dilution in 0.1% BSA in PBS-T. Plates were then washed 3 times with PBS-T and developed by addition of 0.7 mg/mL 2,2′-Azino-bis(3-ethylbenzthiazoline-6-sulfonic acid) diammonium salt (Sigma-Aldrich, St. Louis, MO, USA) and 8.8 mM H_2_O_2_ in 70 mM phosphate-citrate buffer at pH 4.2. The optical density at 405 nm (OD_405_) was measured on an iMark Microplate Absorbance Reader (Bio-Rad, USA). The OD_405_ of control wells containing PBS-T as the antigen was used as background values and subtracted from all test values.

Serum anti-dsDNA antibody titers were measured using a mouse anti-dsDNA ELISA kit (Shibayagi, Shibukawa, Japan) according to manufacturer instructions.

### Immunoblot analysis

Immunoblot analysis was performed as previously described^81^. To preserve phosphorylated Akt protein, immediately after collection from mice under anesthesia, organs were frozen in liquid nitrogen, after which ice-cold TCA/acetone was applied to the frozen tissues for overnight incubation at −80 °C. Three different amounts of each protein sample (8 µg, 4 µg, and 2 µg) were subjected to immunoblot analysis. Signal intensities of Akt and phosphorylated Akt were measured using FC8000 software (Menlo Systems, Newton, NJ, USA). The ratio of phosphorylated Akt to Akt in each individual experiment was calculated, with the mean of six individual experiments analyzed.

### Urinalysis

The concentration of protein in urine from individual mice was determined semi-quantitatively by reagent strips for urine analysis (Albustix, Siemens Healthcare Diagnostics) for 3 consecutive days. A drop of urine was collected on the reagent strip. The indicator range for protein was then, within 10 sec, compared with the color chart on the package for each test, which had six gradings: −, ±, 1+, 2+, 3+, and 4+. Reading was done in daylight and by the same observer, who had normal color vision.

### Statistical analysis

All experiments were performed independently in triplicate at least, and statistical significance was calculated using Student’s unpaired *t*-test or Mann-Whitney U test to examine the differences between the two groups of data. A p value < 0.05 was considered significant. Data displayed in the figures and text represent mean ± standard error (SEM) of representative experiments unless otherwise stated.

## Supplementary information

PKN1 kinase-negative knock-in mice develop splenomegaly and leukopenia at advanced age without obvious autoimmune-like phenotypes

## Data Availability

The datasets generated and/or analyzed during the current study are available from the corresponding author on reasonable request.
