# Supplementary material for: PKN1 kinase-negative knock-in mice develop splenomegaly and leukopenia at advanced age without obvious autoimmune-like phenotypes
Source: Sci Rep. 2019 Sep 27;9:13977. doi: 10.1038/s41598-019-50419-2 (PMC6764976; doi:10.1038/s41598-019-50419-2)
Supplement: Supplementary file 1 — PKN1 kinase-negative knock-in mice develop splenomegaly and leukopenia at advanced age without obvious autoimmune-like phenotypes [file 41598_2019_50419_MOESM1_ESM.pdf]

Supplementary Information

PKN1 kinase-negative knock-in mice develop splenomegaly and leukopenia at advanced age without obvious autoimmune-like phenotypes

Salman Mahmud Siddique[1], Koji Kubouchi[1], Yuka Shinmichi[2], Nana Sawada[2], Reiko Sugiura[2], Yasushi Itoh[3], Shunsuke Uehara[4], Kanae Nishimura[5], Shunsuke Okamura[5], Hiroyuki Ohsaki[5], Shingo Kamoshida[5], Yusuke Yamashita[6], Shinobu Tamura[6], Takashi Sonoki[6], Hiroshi Matsuoka[7], Tomoo Itoh[8], and Hideyuki Mukai[1,9]*

[1]Graduate School of Medicine, Kobe University, Kobe, 650–0017, Japan

[2]Laboratory of Molecular Pharmacogenomics, School of Pharmaceutical Sciences, Kindai University, 3-4-1, Kowakae, Higashi-Osaka, 577- 8502, Japan.

[3]Department of Pathology, Shiga University of Medical Science, Otsu, Shiga, Japan.

[4]Department of Biochemistry, Matsumoto Dental University, Shiojiri, Nagano 399-0781, Japan.

[5]Laboratory of Pathology, Department of Medical Biophysics, Kobe University Graduate School of Health Sciences, 7-10-2 Tomogaoka, Suma, Kobe, Hyogo, 654-0142, Japan.

[6]Department of Hematology/Oncology, Wakayama Medical University, Wakayama, Japan.

[7]Division of Medical Oncology and Hematology, Kobe University Hospital, Kobe, Hyogo, Japan.

[8]Department of Diagnostic Pathology, Kobe University Hospital, Kobe, Hyogo 650-0017, Japan.

[9]Biosignal Research Center, Kobe University, Kobe, 657- 8501, Japan.

*Correspondence and requests for materials should be addressed to H.M. (email: mukinase@kobe-u. ac.jp)

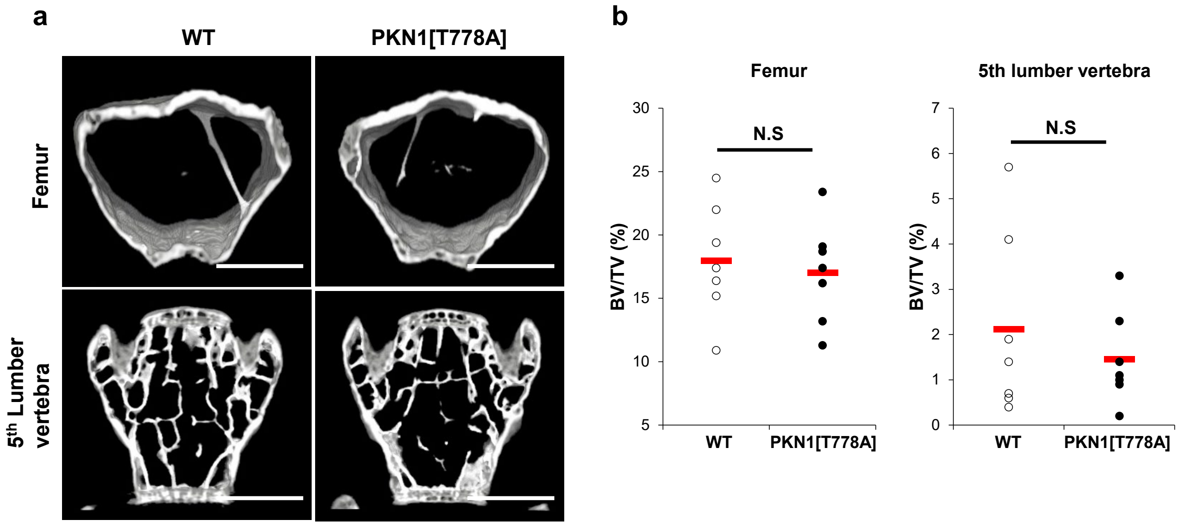

Supplementary Figure S1. Analysis of bone morphology. (A) Micro-CT analysis (ScanXmate-A080, Comscan Tecno) was performed to measure morphological indices in the femurs and fifth lumbar vertebrae. In Femur, these indices were calculated in trabecular bones located between 0.5 and 1.5 mm from the growth plates using image analysis software (TRI/3D-BON, Ratoc System Engineering). Morphological indices of fifth lumbar vertebrae were calculated in trabecular bones located between 0.76 and 0.9 mm from the ventral surface of the vertebral body. Scale bar 2 mm. (B) the ratio of the volume of cancellous bone to the volume of the whole analysis site was calculated as Bone volume / Tissue volume (BV / TV; %) and compared between WT and PKN1[T778A]. Data were analyzed by unpaired *t*-test. n = 7. N.S, not significant.

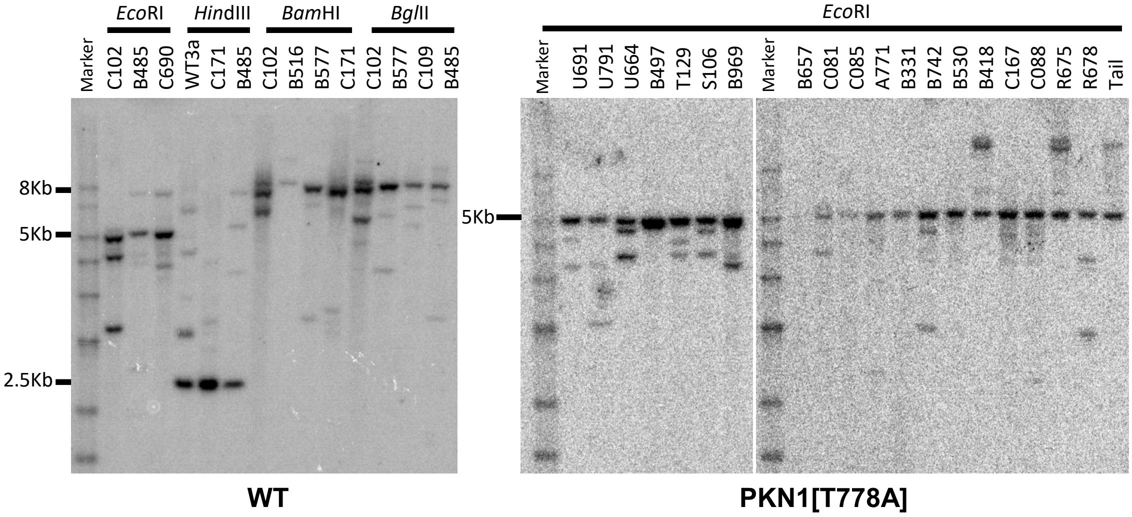

Supplementary Figure S2. Southern blot analysis of splenic DNA probed with pMJH4 probe. Representative image shows rearrangement of *Igh* gene J region in WT samples with indicated restriction enzymes and PKN1[T778A] samples with *Eco*RI. Images of PKN1[Y778A] samples are taken from 2 different gels. Full images are shown in Supplementary Figure S6. Germ line bands are at 5 kbp for *EcoR*I, 2.5 kbp for *Hin*dIII, 8 kbp for *Bam*HI and *Bgl*II.

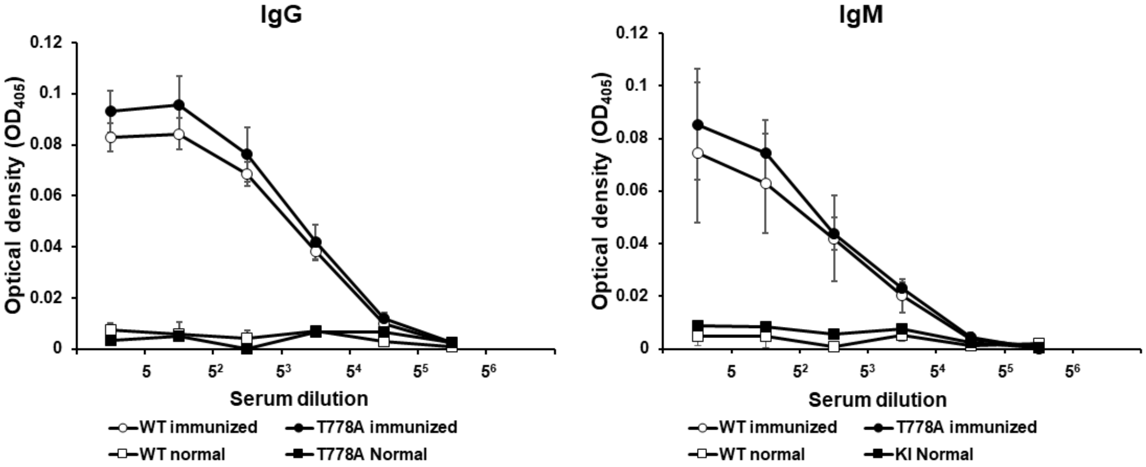

Supplementary Figure S3. Measurement of ovalbumin specific IgG and IgM titers by ELISA in immunized WT and PKN1[T778A] mice serum. The data are presented as mean OD_{405} ± SEM of six mice per group at various dilution points.

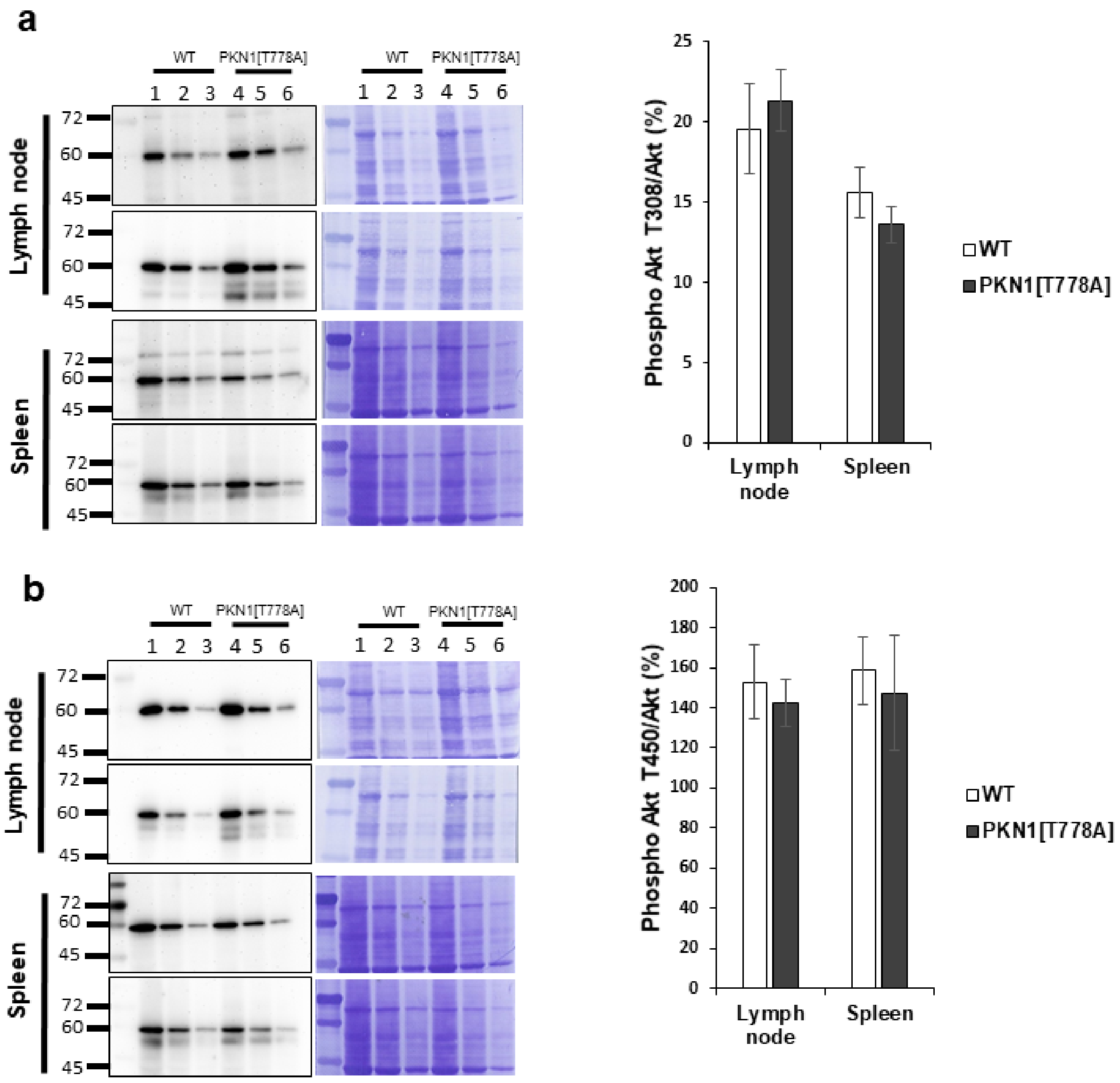

Supplementary Figure S4. Measurement of phosphorylation of Akt at (a) T308 and (b) T450. Representative images of immunoblotting (cropped images) and quantification of Akt phosphorylation at T308 and T450 position (relative to total Akt protein) are shown. Full length images are shown in Supplementary Figure S7. Data represents average of three individual experiments. Data were analyzed by unpaired *t*-test. Lane 1-3: WT samples; 1X,

2X and 4X dilutions. Lane 4-6: PKN1[T778A] samples; 1X, 2X and 4X dilutions.

Molecular weight of Akt and P-Akt are 59 KDa and 60 KDa respectively.

Supplementary Figure S5. Uncropped scans.

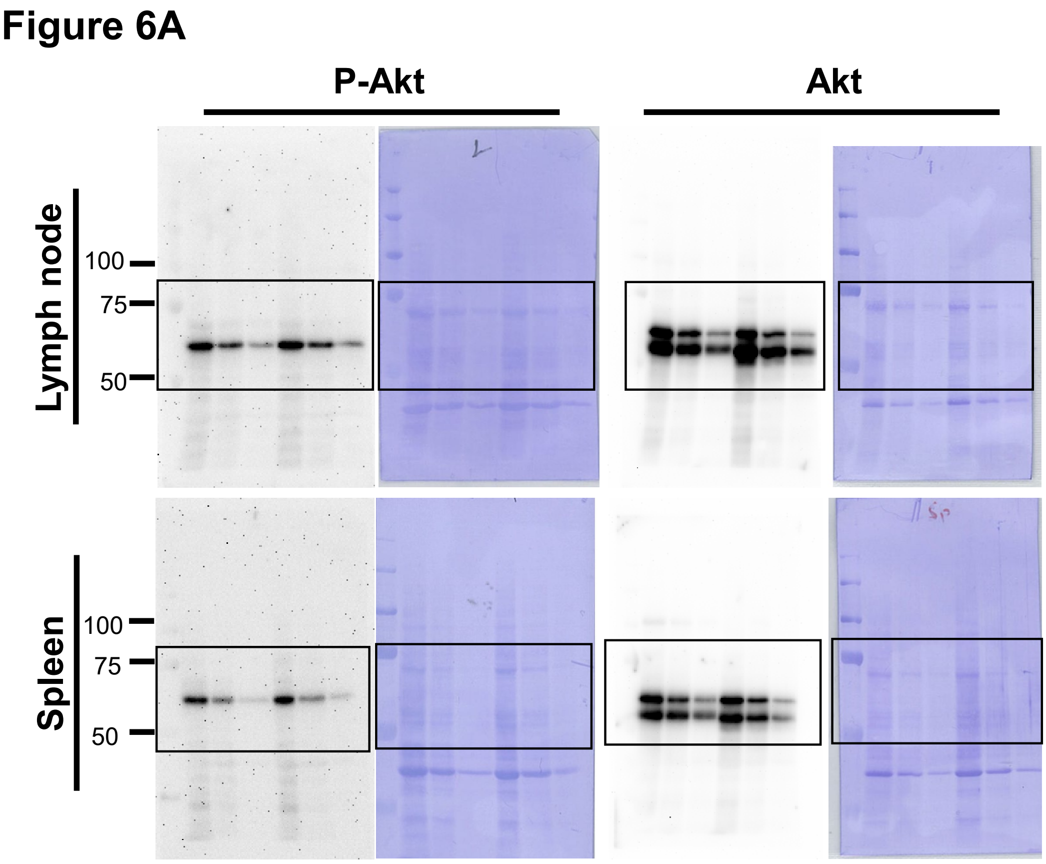

Supplementary Figure S6. Uncropped scans

Supplementary Figure S2

WT samples digested with indicated restriction enzymes

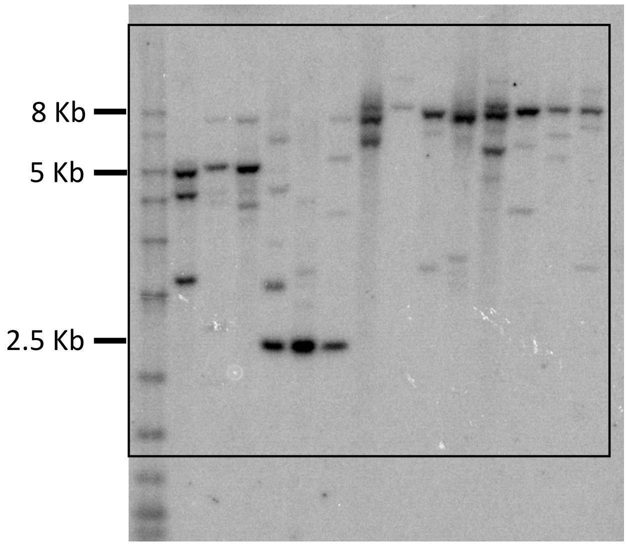

PKN1[T778A] samples digested with *Eco*RI

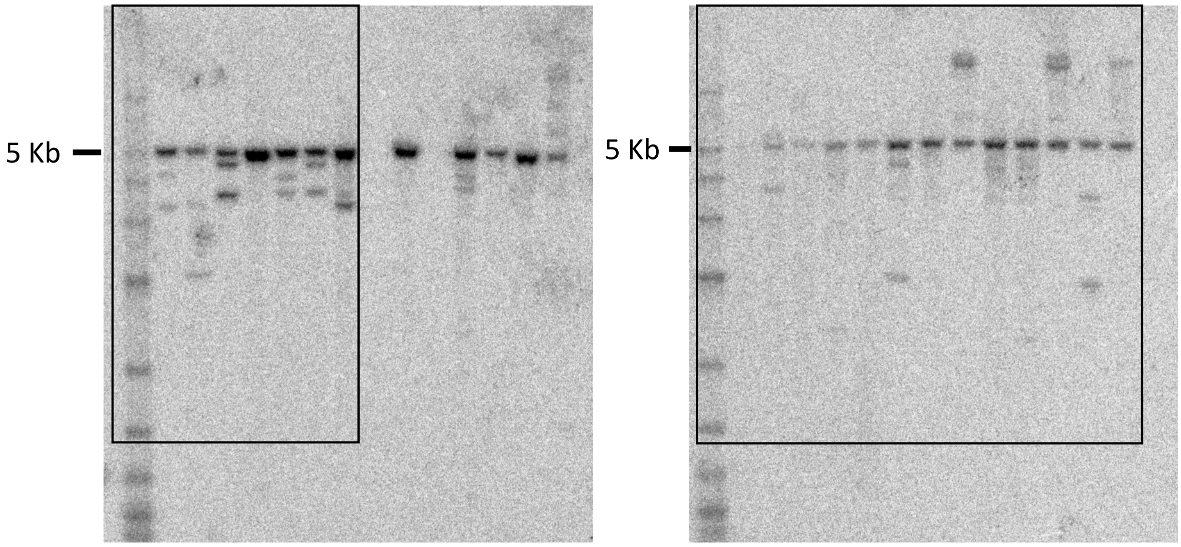

Supplementary Figure S7. Uncropped scans.

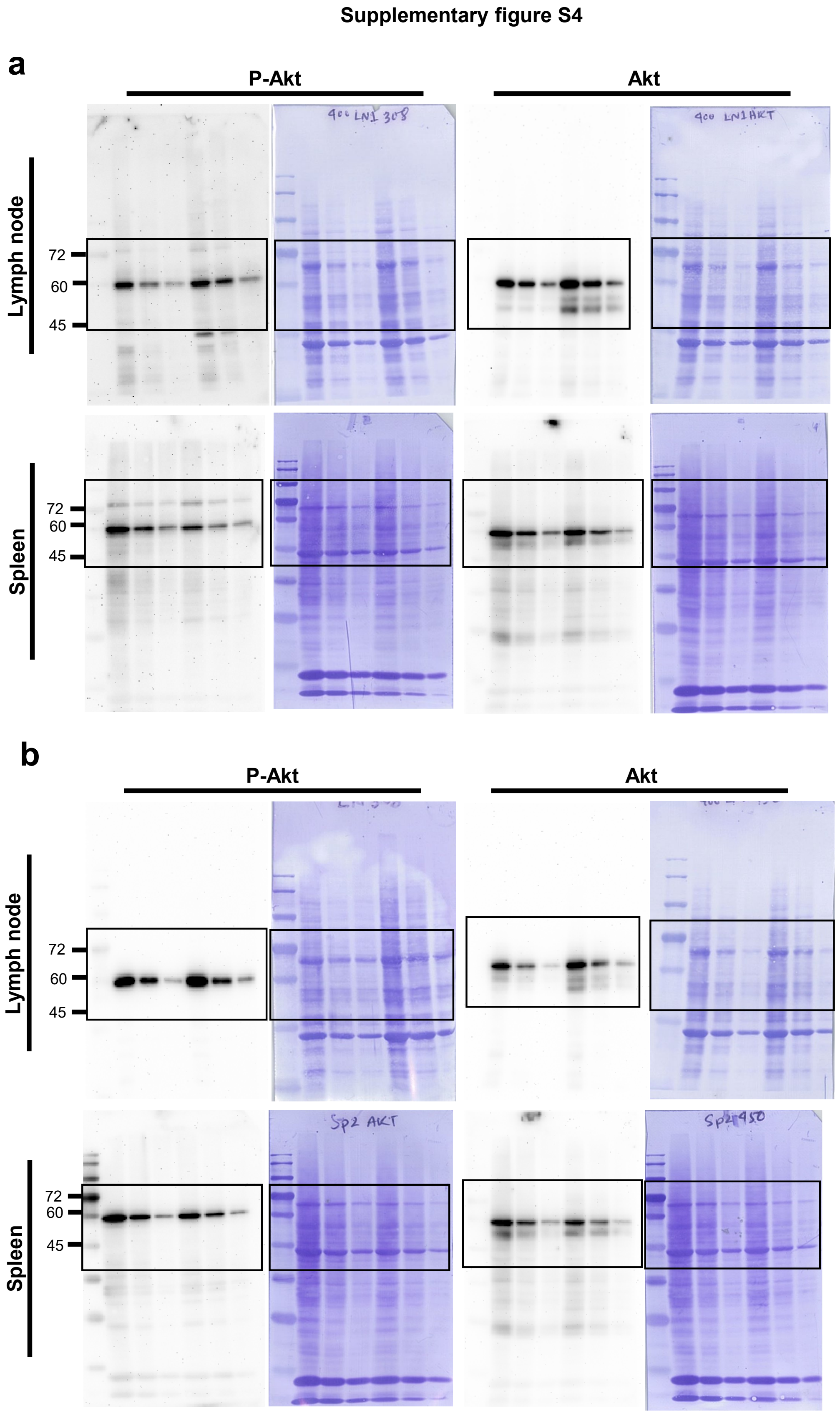

Supplementary Table S1. Summary of histological findings from the spleen of aged WT mice. W, week; Sp.wt., spleen weight; HE, Haemotoxylin and Eosin; WP, white pulp; RP, red pulp.

Mouse ID.	Basic information			HE features (spleen)
	Age (W)	Gender	Sp.wt. (g)	
**C102**	64	F	0.91	Structure distorted, WP expanded, RP reduced, megakaryocytes less
**B522**	96	F	0.9	Structure distorted, expanded RP, many megakaryocytes
**B516**	94	M	0.9	Follicles maintained, WP expanded, RP reduced, less megakaryocytes
**B577**	92	F	0.63	Follicles maintained, a little expanded RP, many megakaryocytes.
**C109**	64	F	0.43	Structure distorted, WP expanded, RP reduced, less megakaryocytes
**WT 1a**	98	F	0.40	Follicles maintained, RP expanded, many megakaryocytes
**B200**	110	M	0.39	Follicles maintained, many megakaryocytes
**B471**	94	M	0.27	Structure distorted, many megakaryocytes
**WT 3a**	98	F	0.22	Follicle maintained, increased small WP, many megakaryocytes
**B116**	118	M	0.19	Structure distorted, many megakaryocytes
**C171**	61	F	0.18	Structure distorted, WP expanded, RP reduced, very few megakaryocytes
**C069**	67	F	0.14	Follicles maintained, WP expanded, less megakaryocytes
**B617**	90	F	0.13	Follicle maintained, less megakaryocytes
**C690**	70	F	0.13	Follicle maintained, WP relatively expanded, less megakaryocytes
**B780**	76	M	0.13	Follicles a little distorted, many megakaryocytes
**S287**	74	F	0.12	Normal
**WT-R**	55	M	0.11	Normal
**WT-S**	67	M	0.1	Normal
**WT-3**	67	M	0.1	Normal
**WT-T**	66	M	0.08	Normal
**S107**	90	M	0.08	Normal
**S165**	92	F	0.07	Normal
**WT-2**	98	F	0.06	Normal

Supplementary Table S2. Summary of histological findings from the spleen of aged PKN1[T778A] mice. W, week; Sp.wt., spleen weight; HE, Haemotoxylin and Eosin; WP, white pulp; RP, red pulp.

Mouse ID	Basic information			HE features (spleen)
	Age (w)	Gender	Sp.Wt. (g)	
**U793**	85	F	3.1	Structure lost, no definite marginal zone, monotonous pattern of cell distribution, less megakaryocytes
**U227**	76	F	1.54	Structure distorted, increased cellularity with moderate number of megakaryocytes
**U791**	89	F	1.23	Structure relatively distorted, WP expanded with increased cellularity and many megakaryocytes
**U664**	88	F	1	Structure relatively distorted, RP expanded with increased cellularity and megakaryocytes
**U702**	84	F	0.9	Structure lost, monotonous pattern of cell distribution.
**U690**	79	F	0.82	Structure relatively distorted, WP expanded, increased cellularity and megakaryocytes in the RP
**U691**	98	F	0.81	Structure relatively distorted, RP expanded with increased cellularity and megakaryocytes
**T433**	104	F	0.80	Follicle maintained, RP expanded with increased cellularity and many megakaryocytes
**T129**	81	F	0.75	Structure distorted, WP expanded, increased RP cellularity with many megakaryocytes
**S106**	90	M	0.68	Structure a little distorted, WP expanded, moderate number of megakaryocytes
**B969**	74	F	0.62	Structure distorted, WP expanded, RP shrunk, less megakaryocytes
**B723**	82	M	0.51	Structure relatively distorted, expansion of RP with many megakaryocytes
**B657**	76	F	0.33	Structure relatively distorted, WP expanded, increased small follicles, less megakaryocytes
**C165**	61	F	0.32	Structure relatively distorted, expansion of RP with increased cellularity, many megakaryocytes
**B416**	98	M	0.29	Follicle maintained, increased RP cellularity with many megakaryocytes
**B529**	84	F	0.29	Follicle maintained, increased RP cellularity with many megakaryocytes
**B970**	74	F	0.27	Structure relatively distorted, increased cellularity, many megakaryocytes.
**B415**	98	M	0.23	Structure a little distorted, increased cellularity with many megakaryocytes
**B742**	76	M	0.2	Follicle maintained. Increased and expansion of WP, moderate number of megakaryocytes
**B530**	84	F	0.19	Structure relatively distorted, WP expanded, RP reduced, few megakaryocytes
**B418**	89	F	0.19	Structure a little distorted, WP expanded, RP reduced, less megakaryocytes
**C167**	61	F	0.19	Structure a little distorted, WP expanded, increased cellularity with moderate number of megakaryocytes
**C088**	65	F	0.18	Follicle maintained, increased cellularity with moderate number of megakaryocytes

Supplementary Table S3. Red blood cell indices of aged mice. RBC, red blood cell; HGB, hemoglobin; HCT, hematocrit; MCV, mean corpuscular volume; MCH, mean corpuscular hemoglobin; MCHC, mean corpuscular hemoglobin concentration. n = 6.

	**WT**	**PKN1[T778A]**
**RBC [10^4/uL]**	1309 ± 44	1281 ± 33
**HGB [g/DL]**	18.2 ± 0.4	17.7 ± 0.4
**HCT [%]**	59.2 ± 1.5	57.0 ± 1.0
**MCV [fL]**	226.2 ± 1.9	222.3 ± 1.9
**MCH [pg]**	69.6 ± 1.0	68.9 ± 0.6
**MCHC [g/DL]**	153.6 ± 1.1	155.2 ± 1.5

(mean ± SEM)

Supplementary Table S4. Urine protein data from aged WT and PKN1[T778A] mice tested by Albustix for 3 consecutive days.

	Mouse information			Urine protein level		
	ID	Age (weeks)	Gender	Day - 1	Day - 2	Day - 3
PKN1[T778A]	S282	80	M	1+	1+	±
	S283	80	M	±	1+	1+
	S544	52	M	1+	1+	1+
	S545	52	M	2+	1+	1+
	B398	100	M	±	±	±
	B526	92	M	1+	1+	1+
	B741	76	M	1+	1+	±
	B742	76	M	1+	1+	1+
	B743	76	M	1+	1+	±
	B745	76	M	1+	1+	1+
	B773	75	M	2	1+	1+
	B774	75	M	3+	3+	3+
	B808	65	M	1+	1+	1+
	B809	65	M	1+	1+	1+
WT	WT1	55	M	2+	1+	1+
	WT2	55	M	1+	1+	±
	WT3	55	M	1+	1+	±
	WT4	55	M	±	1+	±
	S165	92	F	±	±	±
	B701	72	M	1+	1+	1+
	B702	72	M	1+	1+	1+
	B785	76	M	1+	1+	1+
	B470	94	M	1+	1+	1+
	B471	94	M	1+	1+	1+
	B472	94	M	1+	±	1+
	B510	96	M	±	±	1+
	B512	96	M	1+	±	1+
	B514	96	M	1+	±	1+
	B516	96	M	1+	1+	1+
